# Values associated with Nordic Walking: An international cross-sectional survey among individuals practicing walking with poles

**DOI:** 10.1371/journal.pone.0314171

**Published:** 2024-12-27

**Authors:** Wioletta Szymczak, Krzysztof Jurek, Monika Dobrogowska

**Affiliations:** Department of Sociology of Culture, Religion and Social Participation, John Paul II Catholic University of Lublin, Lublin, Poland; Universidad Santiago de Cali, COLOMBIA

## Abstract

**Introduction:**

The article is devoted to the sociological exploration of the social phenomenon that Nordic Walking has become in Europe and worldwide over the recent decades.

**Aim:**

It is based on the results of original international sociological research study on the issue of sociodemographic profiles of Nordic Walkers in the context of the values associated with this sports activity.

**Methods:**

A cross-sectional quantitative study with convenience sampling study was performed among 416 Poles, 132 Europeans from 11 countries (Germany, the UK, Spain, France, Italy, Portugal, Ireland, Denmark, Austria, Sweden, and Norway), and 212 participants from 5 non-European countries (the USA, Canada, Australia, New Zealand, and Japan). The study used an author’s tool that included questions concerning the ways of doing Nordic Walking and its and social and organizational contexts, its classification and interpretation as a health-related, recreational, or sports activity, its position in the space of everyday life and in the sphere of preferred values, motives for walking with poles, meanings attributed to this activity, and the individual and social benefits associated with it.

**Results:**

The values most often associated with practicing Nordic Walking belong to the personal and psychophysical spheres. Their dominant position suggests that this activity is a complex one and that its advantages are not restricted to its motor aspects or to contact with nature. It is not a simple activity of walking with poles, as the initial stereotypes seem to suggest, but a kind of movement with a defined framework and a set of principles, engaging the physical and personal spheres of those who practice it, having a cultural meaning, and marked by cultural rootedness.

**Conclusion:**

The research results can be useful for health care experts and for those responsible for building prevention strategies in various social groups. This is because they draw attention to the category of accessible and at the same time effective activities, with high individual and social potential. At the same time, they show that an important element of promotion strategies and social campaigns aimed at popularizing sport and healthy lifestyle should be axiological categories as significant predictors of individuals’ actions and decisions.

## Introduction

The article is devoted to the sociological exploration of the social phenomenon that Nordic Walking has become in Europe and worldwide over the recent decades. It is based on the results of original international sociological research study, and concerns an issue that has not been analyzed in this manner before in the context of the social phenomenon in question. Our interest is focused on individuals engaging in this form of physical activity, their sociodemographic characteristics, and the meanings attributed to the activity itself. Placing these explorations in the cultural context, we assumed that Nordic Walking carried implicit references to values and that the axiological perspective was one of the keys to identifying and explaining the differences between pole walkers. It was in this context that we posed a question about the profiles of individuals who practiced Nordic Walking, amounting to configurations of values attributed to this activity and walkers’ sociodemographic characteristics. The text refers to the issues and findings of scientists who interpret Nordic Walking as a sport polymorphous, being a health, universal, recreational, free-time, sports, prosocial, and integrative activity.

Since the late 1990s, when “walking with poles” began to gain popularity in Europe [1–4] and worldwide [5], the range of dimensions and identifiable individual and social benefits linked with it has been growing as well. In the first phase of the development, popularization, and scientific analyses of Nordic Walking, prominence was given to its health aspects, characteristic of every physical activity and associated with providing stimuli indispensable for normal functioning, improving health condition, enhancing quality of life, and preventing the processes of aging [6]. At the same time, scholars explicated characteristics specific to this type of exercise, as distinct from walking, running, cycling, etc. The health outcomes of Nordic Walking are located in the following areas: improvement in the functioning of the cardiovascular and respiratory systems, improvement in the efficiency of locomotive organs and motor coordination, cancer and obesity treatment, depression relief, the regenerating value of group walking in the bosom of nature, and recently also convalescence after COVID-19 [7–14]. Researchers have been exploring the associations of this kind of motor activity with quality of life and psychological well-being; as far as the sports dimension of well-being is concerned, explorations have addressed the effect of fast pole walking on sportspeople and amateurs [15] and its overall effect on the physical function of the human organism [16–18]. An important area, highlighted from the beginning, is prevention [19–21]. There are many studies that provide evidence of the positive impact of walking with poles on health. Nordic Walking could be a useful as aerobic training modality for weight control and cardiorespiratory fitness [22]. Nordic walking programs offer an effective way to reduce pain and fatigue in individuals with chronic conditions. Its simplicity and ease of learning make it suitable for a wide range of participants, potentially leading to better adherence and long-term benefits [23]. The positive effects of walking with poles are visible in the case of many diseases. Nordic Walking could serve as a novel, community-based therapeutic approach for asthma patients, offering an affordable and feasible option. When combined with education and standard care, Nordic walking may enhance exercise tolerance and improve other asthma-related outcomes [24].

A distinctive feature of the activity discussed in this article is its universality, conveyed by the term used in social sciences: “a sport for everyone i.e. people of equal age, with different physical or health conditions [5, 25–27]. Nordic Walking can be practiced by anyone, anywhere, and anytime [28, 29], in various natural conditions [30], not only on an amateur and recreational basis but also as sports competition, as shown by the now regularly held national and international cup tournaments and European or world championships [31]. Poland is a leader in initiating and organizing these events. The accessibility of Nordic Walking does not require large financial outlays (gear, sportswear), large amounts of time, or special training sites. It is considered a safe and easy form of physical activity, practicable for nearly everyone [2, 29].

The accessibility and ease of practicing Nordic Walking implies its social potential: inclusive and integrative, related by researchers to different forms of sport [32]. This is because its popularization means the activation of meritocratic thinking, which does not change the fact that empirical knowledge confirms the impact of various sociodemographic determinants [33]. The inclusive, integrative, and development-stimulating character of Nordic Walking manifests itself in a diverse cross-section of training groups, with participants varied in terms of age, physical fitness, and skills, living in cities and villages, representing different occupations, financial statuses, and social positions. Among other things, these facts imply strengthening social cohesion and inspiring the development of local communities [34].

Nordic Walking is also classified into and explored as belonging to the sociological categories of free time and recreational sport [19, 35]. This is because it allows for making active use of leisure time, which constitutes an element of what is called healthy lifestyle [36, 37], enables maintaining social relations thanks to interactions with other walkers, acquaintances, friends, family members, and contestants through communing with nature and outdoor exercise. Regarded as an all-year-round activity, it requires no special weather conditions.

Moreover, sociologists point out that it has the advantages significant in every sports activity: competition, leisure and recreation, quasi-festive experience, the regularity requirement, stamina training, opportunities to develop effort and fitness habits, and a chance to assimilate the principles of *fair play*, solidarity, and support [38–40]. To use concepts from the sociology of sport again, Nordic Walking can be defined as an amateur and recreational sport with a dynamically developing professional branch. The thesis that Nordic Walking is a sport for everyone is supported by the results of empirical studies conducted in different scientific disciplines, which identify the sociodemographic characteristics of pole walkers. In empirical research, respondents are differentiated according to variables investigated in health sciences or social sciences, such as age, gender, education, place of residence, perceived financial situation, health condition, and physical fitness. The existing findings in social sciences indicate that Nordic Walking is more often practiced by women, individuals with higher education, people aged 20 to 60 years or even older, those associating this activity with the value of health and appreciating its effect on their psychophysical sphere, and economically active inhabitants of big cities. The findings of British, Australian, and Polish sociologists show, however, that demographic data are not a frequent element of research reports [2, 4, 6, 20, 30, 36, 37].

The activity in question can be explained within the framework of different paradigms. The assumption that the structure of sport comprises subjective-personal, ideological-normative, and material elements [41] means that an important issue in the analysis of sports behaviors is their associations with values. The axiological perspective used in the interactive way of explaining the actions of individuals and society and in explaining their life-worlds as worlds of culture, also applied in the sociology of sport–relies on the meanings attributed to actions by social actors and on the interactions based on them [19–44]. The voluntary participation in sport, membership in sport-related groups, associations, and organizations, and recreational or organized practice of sports is also interpreted as form of democratic engagement, an important element of which is axiological references [45, 46].

Nordic Walking embodies values typical of every sports activity: autotelic (sport as a value) or instrumental: personal values, vital values, and hedonistic and agonistic values; prosocial values; ethical and moral values (e.g., responsibility, the *fair play* principle); socioeconomic values; aesthetic values; art-related values; religious and sacred values (implicitly or explicitly determining contestants’ attitudes and supporting their efforts; sport as a “civil religion”); and the value of peace (emphasized in the Olympic movement). Practicing sports and being a sports fan have also been explored in the context of values such as patriotism, politics, sportspeople’s rights and sport-related prudence, justice, fortitude, moderation, caution, truthfulness, discipline, and magnanimity [47–70].

The existing empirical studies on Nordic Walking show that respondents associate it primarily with the values of health and well-being and with the pleasure that is implicitly part of physical activity, outdoor exercise, and communing with nature. They explicate the significance of a sense of belonging to a group, social and emotional support connected with the emergence of close relationships in walking and training groups, and the values of comradeship and friendship [1, 26, 36, 37, 40]. Like other sports activities, the practice of Nordic Walking depends on the position it occupies in each person’s system of values, on the values attributed to this particular exercise, and on sociodemographic and cultural determinants such as gender, health condition, financial status, education, place of residence and the related patterns of spending free time, worker migrations and the increasing number of people doing office work or employed in services, quality of life, and social prestige [62, 71].

The conclusions formulated by scholars and observations concerning the dynamics of the phenomenon led us to hypothesize that the socio-demographic profiles of individuals practicing Nordic Walking are linked to their value choices regarding this physical activity. We assumed that the choice of values usually associated with it defined the individual engagement axis (an axis focusing and organizing action) [46]. The focus of the analyses on values made it possible to consider Nordic Walking in the context of issues important for the individual, and the variables included in the study captured the diversity of respondents. The current explorations center around the questions of who Nordic Walkers are and what configurations of axiological and sociodemographic factors differentiate this group of people. The decision to analyze the values associated with Nordic Walking by different categories of respondents locates our investigations on the cultural plane [72].

### Participants and procedure

We conducted the sociological research in 2022 using an online survey tool (Lime Survey). We applied convenience sampling, administering the survey to Nordic Walkers in Poland and abroad. In the initial phase, we offered participation to individuals from all voivodeships (main administrative units) of Poland and to participants in international Nordic Walking competitions whose names were included in openly accessible lists of contestants. Invitations to complete the survey were also sent to representatives of local and regional training groups and Nordic Walking associations. Through the contacts thus obtained, we reached further respondents and groups. We distributed the survey exclusively via email and through direct contacts, each time requesting the recipient not to make it available in any group, forum, and the like. The survey was to be completed once only. In total, the study included 416 Poles, 132 Europeans from 11 countries (Germany, the UK, Spain, France, Italy, Portugal, Ireland, Denmark, Austria, Sweden, and Norway), and 212 participants from 5 non-European countries (the USA, Canada, Australia, New Zealand, and Japan). The recruitment spanned three months, from 01/02/2022, to 31/07/2022.

### Measure

For the purposes of the research project, we developed a survey questionnaire consisting of several thematic blocks. It included questions concerning the ways of doing Nordic Walking and its and social and organizational contexts, its classification and interpretation as a health-related, recreational, or sports activity, its position in the space of everyday life and in the sphere of preferred values, motives for walking with poles, meanings attributed to this activity, and the individual and social benefits associated with it. The questions covered four areas personal development and self-realization (Cronbach’s alpha = 0.909; sample statements: developing positive/desirable traits of character; cheerful attitude to the world; being able to think about everyday problems/difficulties); health and fitness (Cronbach’s alpha = 0.727; sample statements: preventive effect on my health; positive influence on my figure; reduction of various health problems), satisfaction and well-being (Cronbach’s alpha = 0.772; sample statements: improved sense of well-being lasting longer; feeling of oxygenation and improvement; the prospect of feeling good after training) and ties and community (Cronbach’s alpha = 0.807; sample statements: enjoyment of meeting friends; meetings, conversations while walking; building or strengthening relationships with others). Additionally, the research tool was consulted with specialists and experts in the field of Nordic Walking (including practitioners, trainers, and mentors) and researchers specializing with the indicated issues. In the present study, we considered selected values belonging to the categories indicated in the introduction, associated with sport and at the same time most associated by respondents with Nordic Walking (Table 1). The survey also included questions about sociodemographic variables: age, gender, education, place of residence, financial situation, and quality of life. Additionally, respondents were asked to indicate their preferred forms of practicing Nordic Walking (Table 2).

**Table 1:** Values most associated with practicing Nordic Walking.

Value	*n*	%
Sports success	46	6.1
Mutual support and solidarity	206	27.1
Professionalism (technique)	154	20.3
Health	624	82.1
Fair play	72	9.5
Harmony of body and spirit	344	45.3
Personal growth	188	24.7
Self-discipline	196	25.8
Working on oneself and overcoming weaknesses	280	36.8
Respect for other contestants	40	5.3
Total	2150	-

**Table 2:** Sample characteristics.

Characteristic		Total		Poland		Europe		Other counties	
		*n*	%	*n*	%	*n*	%	*n*	%
**Age**	≤34	66	8.7	38	9.1	24	18.2	4	1.9
	35–45	114	15.0	104	25.0	6	4.5	4	1.9
	46–55	194	25.5	148	35.6	32	24.2	14	6.6
	56–65	186	24.5	80	19.2	30	22.7	76	35.8
	66–75	160	21.1	46	11.1	38	28.8	76	35.8
	>75	40	5.3	-	-	2	1.5	38	17.9
**Gender**	Male	188	24.7	114	27.4	44	33.3	30	14.2
	Female	572	75.3	302	72.6	88	66.7	182	85.8
**Education**	Elementary	2	0.3	2	0.5	-	-	-	-
	Basic vocational	34	4.5	16	3.8	14	10.6	4	1.9
	Secondary	148	19.5	100	24.0	22	16.7	26	12.3
	Higher	576	75.8	298	71.6	96	72.7	182	85.8
**Place of residence**	City	606	79.7	296	71.2	118	89.4	192	90.6
	Village	154	20.3	120	28.8	14	10.6	20	9.4
**Financial situation**	Very good	108	14.2	28	6.7	12	9.1	68	32.1
	Good	394	51.8	222	53.4	66	50.0	106	50.0
	Average	244	32.1	160	38.5	46	34.8	38	17.9
	Poor	14	1.8	6	1.4	8	6.1	-	-
**Quality of life**	Very good	204	26.8	68	16.3	30	22.7	106	50.0
	Good	462	60.8	274	65.9	88	66.7	100	47.2
	Average	84	11.1	72	17.3	6	4.5	6	2.8
	Poor	10	1.3	2	0.5	8	6.1	-	-
**Forms of practicing Nordic Walking**	Mostly individually	240	31.6	122	29.3	34	25.8	84	39.6
	Mostly with friends who also like this form of activity	246	32.4	124	29.8	26	19.7	96	45.3
	Usually with a Nordic Walking instructor and a group	274	36.1	170	40.9	72	54.5	32	15.1
**When do you do Nordic Walking?**	Seasonally	168	22.1	118	28.4	32	24.2	18	8.5
	All year round	592	77.9	298	71.6	100	75.8	194	91.5
**In what way do you do Nordic Walking?**	Recreationally	372	48.9	184	44.2	60	45.5	128	60.4
	As a sport	62	8.2	12	2.9	30	22.7	20	9.4
	Recreationally and as a sport	326	42.9	220	52.9	42	31.8	64	30.2

### Ethical considerations

The research was conducted in accordance with the ethical principles set out in Recommendations from the Association of Internet Researchers. Participation in the study was voluntary and anonymous. All respondents gave their informed consent to participate in the survey online. The informed consent page explained the purpose and method of the study. We obtained consent for study by asking participants to select a mandatory consent item before proceeding with the study. The procedure was approved by the Ethics Committee of the Institute of Sociological Sciences of the John Paul II Catholic University of Lublin (Project identification code: 02/DKE/NS/2022).

### Statistical analysis

The dependent variables–values most associated with practicing Nordic Walking–are dichotomous (1 = the value was selected; 0 = the value was not selected). To determine the predictors of respondents’ choice of values most associated with Nordic Walking, we performed a logistic regression analysis. The predictive value of the models was assessed based on a chi-square test (using the Omnibus Tests of Model Coefficients) and the Hosmer–Lemeshow goodness-of-fit test. Nagelkerke’s *R*^2^ was also calculated. *P* values < .05 were considered statistically significant. The Hosmer–Lemeshow goodness-of-fit statistic consists in grouping observations into risk deciles and comparing the observed likelihood with the expected likelihood within each decile. If the Hosmer–Lemeshow test statistic is higher than 0.05, the null hypothesis is adopted stating that there is no difference between the predicted and the expected scores on the variables, which means that the model is correct. The Nagelkerke coefficient, corresponding to fit index in the classic linear regression model, is a modification of the Cox and Snell index. Its values range between 0 and 1. All statistical analyses were performed using IBM SPSS Statistics for Windows, Version 26.0 (released in 2019 by IBM Corp., Armonk, NY, USA).

## Results

Table 2 presents the sociodemographic and other characteristics of the sample, divided into Poland, Europe, and other countries. Respondents’ age ranged between 18 and 94 years, with a mean age of *M* = 55.15 years (*SD* = 13.54). The majority of the respondents were women (75.3%) and city residents (79.7%). Three-quarters of the sample (75.81%) had higher education.

The most frequently indicated values associated with practicing Nordic Walking were health, harmony of body and spirit, and working on oneself and overcoming weaknesses.

Next, we assessed the effect of sociodemographic variables on the choice of values associated with Nordic Walking. For this purpose, we performed a series of logistic regression analyses. The values that were indicated most often (> 100) were taken into account. The article presents the results for only the three models whose indexes were acceptable. They concerned the following values: “health,” “working on oneself and overcoming weaknesses,” and “personal growth.”.

In the first model, the analyzed value was health (Table 3). The statistics for this model were acceptable, which means the model can be seen as well-fitted, chi^2^ = 92.711, *p* < .001; Nagelkerke’s *R*^2^ = .189. Model accuracy was also verified using the Hosmer–Lemeshow test, chi^2^ = 10.738, *p* = .217. The logistic function correctly predicted belonging to one of two groups in 82.6% of cases. The factors significantly related to the choice of health as the value associated with Nordic Walking were: living in Europe (Exp(B)  =  7.269, 95% CI [3.219, 16.413], *p* < .001) or a non-European country (Exp(B)  =  4.277, 95% CI [2.132, 8.581], *p* < .001), elementary or vocational education (Exp(B)  =  0.271, 95% CI [0.115, 0.641], *p* = .003), financial situation (Exp(B)  =  1.695, 95% CI [1.041, 2.758], *p* = .034), place of residence (Exp(B)  =  0.432, 95% CI [0.247, 758], *p* = .003), very good quality of life (Exp(B)  =  0.304, 95% CI [0.133, 0.691], *p* = .005), and the way of practicing Nordic Walking (Exp(B)  =  0.509, 95% CI [0.293, 0.885], *p* = .017). Foreign subjects indicated this value more often than Polish ones. Similarly, individuals reporting an average or poor financial situation pointed to health as a value associated with Nordic walking more often than those in a very good or good financial situation. In the case of respondents with elementary or vocational education (compared to those with higher education), reporting very good quality of life (compared to those with average quality of life), and practicing Nordic Walking seasonally (compared to all-year-round walkers), the likelihood of choosing health as a value associated with this activity was lower.

**Table 3:** Logistic regression analysis for factors associated with the choice of health as a value associated with Nordic Walking.

	B	Wald	*p*	Exp(B)	Lower limit 95%	Upper limit 95%
>75 (reference)						
≤34	-0.025	0.001	.972	0.975	0.242	3.934
35–45	-0.007	0.000	.992	0.993	0.256	3.851
46–55	0.557	0.675	.411	1.745	0.462	6.589
56–65	0.970	2.148	.143	2.639	0.721	9.661
66–75	0.799	1.495	.221	2.223	0.618	7.995
Poland (reference)						
Europe	1.984	22.786	< .001	7.269	3.219	16.413
Other countries	1.453	16.730	< .001	4.277	2.132	8.581
Gender (male–reference)	-0.302	1.575	.210	0.739	0.461	1.185
Higher (reference)						
Elementary/vocational	-1.306	8.849	.003	0.271	0.115	0.641
Secondary	-0.265	1.053	.305	0.767	0.462	1.273
Place of residence (city–reference)	-0.838	8.584	.003	0.432	0.247	0.758
Financial situation (very good / good–reference)	0.528	4.508	.034	1.695	1.041	2.758
Quality of life (average–reference)						
Very good	-1.192	8.068	.005	0.304	0.133	0.691
Good	-0.479	1.969	.161	0.619	0.317	1.210
Most often with a Nordic Walking instructor and a group (reference)						
Mostly individually	-0.102	0.149	.699	0.903	0.539	1.513
Mostly with friends who also like this form of activity	-0.358	1.936	.164	0.699	0.422	1.158
When do you do Nordic Walking? (all year round–reference)	-0.675	5.724	.017	0.509	0.293	0.885
In what way do you do Nordic Walking? (recreationally–reference)						
As a sport	0.086	0.139	.709	1.090	0.692	1.718
Recreationally and as a sport	-0.826	4.091	.053	0.438	0.197	1.005

The next value significantly associated with Nordic Walking was “working on oneself and overcoming weaknesses” (Table 4). For this model, the analysis yielded the following statistics: chi^2^ = 89.770, *p* < .001; Nagelkerke’s *R*^2^ = .152. Model accuracy was also verified using the Hosmer–Lemeshow test (chi^2^ = 11.413, *p* = .179). The logistic function correctly predicted belonging to one of two groups in 68.2% of cases. The factors significantly related to the choice of working on oneself and overcoming weaknesses as the value associated with Nordic Walking were: living in Europe (Exp(B)  =  0.356, 95% CI [0.209, 0.609], *p* < .001) or a non-European country (Exp(B)  =  0.563, 95% CI [0.349, 0.907], *p* = .018), elementary or vocational education (Exp(B)  =  0.386, 95% CI [0.158, 0.942], *p* = .036), secondary education (Exp(B)  =  0.553, 95% CI [0.362, 0.846], *p* = .006), walking with poles individually (Exp(B)  =  0.541, 95% CI [0.360, 0.814], *p* = .003) or together with friends (Exp(B)  =  0.620, 95% CI [0.414, 0.929], *p* = .021), and doing Nordic Walking as a sport (Exp(B)  =  1.525, 95% CI [1.080, 2.154], *p* = .017). Respondents living abroad (compared to those from Poland), individuals with elementary or vocational and secondary education (compared to those with higher education), and participants doing Nordic Walking individually or with friends (compared to those who usually practiced it with an instructor) less often indicated the value of working on oneself and overcoming weaknesses. In the case of individuals who treated Nordic Walking as a sport, the likelihood of choosing this value was higher.

**Table 4:** Logistic regression analysis for factors associated with the choice of working on oneself and overcoming weaknesses as a value associated with Nordic Walking.

	B	Wald	*p*	Exp(B)	Lower limit 95%	Upper limit 95%
>75 (reference)						
≤34	-0.166	0.106	.745	0.847	0.312	2.298
35–45	0.095	0.038	.845	1.100	0.425	2.844
46–55	-0.667	2.044	.153	0.513	0.206	1.281
56–65	-0.123	0.080	.778	0.885	0.378	2.072
66–75	-0.138	0.099	.753	0.871	0.369	2.057
Poland (reference)						
Europe	-1.032	14.471	< .001	0.356	0.209	0.606
Other countries	-0.575	5.576	.018	0.563	0.349	0.907
Gender (male–reference)	-0.246	1.529	.216	0.782	0.529	1.155
Higher (reference)						
Elementary/vocational	-0.952	4.377	.036	0.386	0.158	0.942
Secondary	-0.592	7.479	.006	0.553	0.362	0.846
Place of residence (city–reference)	0.002	0.000	.993	1.002	0.665	1.510
Financial situation (very good / good–reference)	-0.237	1.449	.229	0.789	0.536	1.161
Quality of life (average–reference)						
Very good	-0.328	0.994	.319	0.721	0.378	1.372
Good	-0.362	1.750	.186	0.696	0.407	1.191
Most often with a Nordic Walking instructor and a group (reference)						
Mostly individually	-0.614	8.720	.003	0.541	0.360	0.814
Mostly with friends who also like this form of activity	-0.477	5.363	.021	0.620	0.414	0.929
When do you do Nordic Walking? (all year round–reference)	0.225	1.158	.282	1.253	0.831	1.888
In what way do you do Nordic Walking? (recreationally–reference)						
As a sport	0.422	5.736	.017	1.525	1.080	2.154
Recreationally and as a sport	-0.684	3.080	.079	0.505	0.235	1.083

In the last of the models, we analyzed personal growth as a value significantly associated with practicing Nordic Walking (Table 5). The statistics for this model were as follows: chi^2^ = 46.284, *p* < .001; Nagelkerke’s *R*^2^ = .088. Model accuracy was also verified using the Hosmer–Lemeshow test (chi^2^ = 13.789, *p* = .087). The logistic function correctly predicted belonging to one of two groups in 75.3% of cases. The factors significantly related to the choice of personal growth as a value associated with Nordic Walking were age below 35 years (Exp(B)  =  2.883, 95% CI [1.081, 7.686], *p* = .034) and living in a European country (Exp(B)  =  3.491, 95% CI [1.970, 6.183], *p* < .001). Respondents from Europe (compared to those from Poland) and the youngest participants, up to 34 years of age, more often pointed to personal growth as a value associated with this activity.

**Table 5:** Logistic regression analysis for factors associated with the choice of personal growth as a value associated with Nordic Walking.

	B	Wald	*p*	Exp(B)	Lower limit 95%	Upper limit 95%
>75 (reference)						
≤34	1.059	4.480	.034	2.883	1.081	7.686
35–45	0.623	1.728	.189	1.865	0.736	4.726
46–55	0.380	0.727	.394	1.462	0.611	3.500
56–65	-0.002	0.000	.997	0.998	0.453	2.199
66–75	-0.167	0.167	.683	0.847	0.381	1.883
Poland (reference)						
Europe	1.250	18.361	< .001	3.491	1.970	6.183
Other countries	-0.320	1.184	.277	0.726	0.408	1.292
Gender (male–reference)	0.154	0.482	.487	1.167	0.755	1.802
Higher (reference)						
Elementary/vocational	-0.193	0.157	.692	0.824	0.316	2.148
Secondary	-0.111	0.218	.641	0.895	0.563	1.424
Place of residence (city–reference)	-0.282	1.521	.217	0.754	0.482	1.181
Financial situation (very good / good–reference)	-0.334	2.357	.125	0.716	0.468	1.097
Quality of life (average–reference)						
Very good	-0.026	0.005	.942	0.974	0.479	1.983
Good	-0.080	0.070	.791	0.923	0.512	1.667
Most often with a Nordic Walking instructor and a group (reference)						
Mostly individually	0.010	0.002	.964	1.010	0.651	1.568
Mostly with friends who also like this form of activity	-0.214	0.852	.356	0.808	0.513	1.271
When do you do Nordic Walking? (all year round–reference)	0.370	2.263	.132	1.448	0.894	2.345
In what way do you do Nordic Walking? (recreationally–reference)						
As a sport	-0.016	0.007	.934	0.984	0.675	1.436
Recreationally and as a sport	-0.085	0.054	.816	0.919	0.451	1.874

## Discussion

To the best of our knowledge, this is the first sociological study to explore the sociodemographic characteristics of individuals practicing Nordic Walking and attributing specific values to that activity. The analyses show that the most frequently chosen values include: “health,” “working on oneself and overcoming weaknesses,” and “personal growth.”

The choice of the health value was determined by variables such as country of origin, financial situation, education, quality of life, and the way of practicing Nordic Walking. The health value was more often indicated by respondents from European and non-European countries than by those from Poland. Health as a value is treated as a kind of health capital, an element of individual well-being [73], and the aim of prevention efforts. It can be concluded that health prevention and the awareness of its significance in Poland is in the phase of intense development but has not yet reached a level as high as in other European countries [74]. As mentioned in the introduction, Polish researchers’ interest is mainly in the health dimension of Nordic Walking and, increasingly often, in health prevention but the latter does not yet have the established position in public awareness that it has in Western countries, such as Germany [21, 26, 30, 36]. A context that reveals these differences is campaigns for the popularization and implementation of prevention programs, including sports activities. It is, to a great extent, from this perspective that Nordic Walking has been analyzed [75–77]. Focusing on those countries where prevention has an established place in public policies, it is worth stressing that population aging, the growing demand for medical services, and the increasing costs of these serviced are a challenge for every health care system. They show that orientation towards treatment alone is insufficient, the response to this being health prevention and promotion. With its multifaceted potential, Nordic Walking fits into this trend perfectly [78–81].

Empirical data analyses show that the likelihood of choosing health as a value associated with Nordic Walking is higher in individuals practicing all year round. It can be assumed that those who do Nordic Walking during bad weather conditions are enthusiasts, individuals who prefer systematic training, sportspeople keeping fit through activities such as pole walking, and people aware of the functionality of this kind of training for health, appreciating its advantages in all weathers. All-year-round walkers have a consolidated experience of how this kind of exercise influences their psychophysical condition, which is another possible reason for their adherence to all-year-round training and for associating it with the value of health. The above observations are in line with the results of the qualitative research conducted by Woube [80]. They show that special importance is attributed to regular all-year-round physical activity and that this importance is associated precisely with the health category. On the one hand, this is part of a trend associated with striving to maintain health and physical strength, clearly present in Western society. However, what also plays a significant role is sociocultural factors, such as a desire for competition, a desire to face up to one’s weaknesses, and–finally–holding oneself up as a model for peers to imitate [80].

As shown by the present study, health is more often indicated as a value associated with practicing Nordic Walking by individuals with higher education. This variable is conducive to a healthy lifestyle. It can be assumed that people with higher education will show a greater awareness of the significance of sport for health and prevention. Empirical studies suggest that higher education is positively related to physical activity. In 2018 in Poland, practicing sports was reported most often by respondents with higher education (83%) [81]. Kari and colleagues present evidence showing that education can be a factor leading to greater free-time physical activity and, consequently, a factor linked with the promotion of health behaviors [82]. Likewise, Stankiewicz et al. found that Nordic Walking was chosen by individuals living in big cities, with higher education, and doing creative work [37].

The respondents who pointed to health as a value associated with practicing Nordic Walking were those in an average or poor financial situation and with average quality of life. This observation may indirectly confirm the fact that Nordic Walking is an inclusive sport, accessible to everyone also at the awareness level. It is a low-cost activity, which makes it practicable for people at different levels of wealth [83]. It can therefore be presumed that the reasons why less wealthy individuals practice Nordic Walking include the fact that this activity does not require great expenses. On the other hand, in a situation of limited financial resources it becomes an appropriate and feasible health care and prevention method. As reported by Lee et al., it is precisely the availability of sports facilities that physical activity is strongly related to, even in countries as developed as Korea [84]. Since many small towns in Poland lack this kind of infrastructure, Nordic Walking is becoming an accessible form of activity promoted by health specialists and experts.

The next value significantly associated with Nordic Walking is “working on oneself and overcoming weaknesses.” The choice of this value was related to country of origin, higher education, the way of walking with poles, and the identification of Nordic Walking as a sport. Self-improvement and self-realization were also identified as significant motives for practicing Nordic Walking by Soroka et al. [4] According to other findings, physical activities and sports strengthen human will, thus playing an important role in developing self-confidence and character building [85]. Our empirical results show that this value was more often chosen by Poles. In this case, an appropriate interpretative context can be Polish culture linked with religiosity. For many years, its characteristic features have included embeddedness in tradition, rootedness in local communities, and association with the moral sphere [86]. In this context, in Polish society, working on oneself is associated with values such as: asceticism, self-discipline, virtue, gaining fitness, and the activation of the body and the mind (especially the mind) [87]. In Western culture, working on oneself and overcoming weaknesses is linked to the categories of satisfaction with oneself, satisfaction with one’s achievements, and competition. Working on oneself can therefore be regarded as a means of self-improvement and as a process leading, for example, to achieving the expected appearance or figure [88].

Research results indicate that associating Nordic Walking with the value of “working on oneself and overcoming weaknesses” was more frequent among individuals with higher education. It can be assumed that higher education favors orientation towards self-control, self-discipline, and work on oneself understood as self-control, particularly when these are aimed at taking care of one’s health. Presumably, the university years are a time when a person becomes aware of certain needs, acquires regular physical activity habits, and develops self-discipline. This was confirmed by research conducted in 2020 in Turkey [89]. Education thus emerges as a predictor of greater awareness of the significance of and the need for self-discipline and self-development [90].

The choice of the value of “working on oneself and overcoming weaknesses” was more frequent among those respondents who practiced pole walking with an instructor and among those who engaged in this activity as a sport rather than recreationally. This observation shows that practicing Nordic Walking professionally implies the kind of experience typical of any sports activity and a belief in the need to struggle with oneself in order to achieve increasingly good results. The sports nature of the activity and walking with an instructor implicitly involves conscious striving to improve one’s walking technique. It is also associated with making controlled effort–with controlling one’s body both during exercise and in a long-term perspective. It is worth adding that earlier empirical findings, reported by Żurawik [91], unambiguously showed that instructors played a key role in teaching participants the right walking technique, providing support, and motivating them to continually improve.

Another value that respondents pointed to as associated with Nordic Walking was personal growth. The factors that determined the choice of this value were country of origin and age. It was more often chosen by respondents from Western countries. In this context, practicing Nordic Walking can be interpreted as one of the manifestations of the lifestyle characteristic of contemporary Western culture. It is marked by orientation towards self-development, self-acceptance, and self-satisfaction [91], developed in a culture of individualism, differentiation, and deinstitutionalization [92]. As indicated above, Nordic Walking is an activity that, on the one hand, allows individuals to build self-confidence, while on the other it is associated with taking care of one’s health, body, appearance, and vitality [93]. These values are particularly dear to the young generation, including young Europeans. Statistical data show that in developed European countries the percentage of people aged 18–24 who do health-enhancing aerobic and muscle-strengthening physical activities in a typical week is 45.8% in Germany, 43.4% in Sweden, 34.7% in Austria, and 37.4% in Denmark. The corresponding figure for Poland is 14.4% [94]. Smyła points out that the central place in young people’s axiological system belongs to health [95]. Połoczańska-Godek points out that it is not always an autotelic value and that it is also treated as an instrumental one and a tool for self-fulfillment [96]. In our study, we found that it was young people (under 35 years of age) who more often pointed to self-development as a value associated with Nordic Walking. It has been found that physical activity, particularly sport, can positively impact young people’s personal growth, especially its aspects such as self-worth, goal setting, and leadership [97].

The current sociological study was exploratory. It is one of the few that address the issue of sociodemographic profiles of Nordic Walkers in the context of the values associated with this sports activity. We are aware, however, that our survey has several weaknesses. The cross-sectional nature of the study did not allow the establishment of any temporal relationships. Additionally, non-probability sampling technique was applied to recruit the required participants. This undermines the generalizability of its results from the sample to the population.

## Conclusion

The presented results of sociological research indicate that the values most often associated with practicing Nordic Walking belong to the personal and psychophysical spheres. Their dominant position suggests that this activity is a complex one and that its advantages are not restricted to its motor aspects or to contact with nature. It is not a simple activity of walking with poles, as the initial stereotypes seem to suggest, but a kind of movement with a defined framework and a set of principles, engaging the physical and personal spheres of those who practice it, having a cultural meaning, and marked by cultural rootedness. What bears witness to this is the values attributed to Nordic Walking. The identification of the sociodemographic factors behind the choice of these values makes it possible to formulate further significant and illuminating conclusions and their implications, highlighting the importance of the present sociological study.

What supports the above conclusion about Nordic Walking as a physical activity with defined characteristics and professional attributes is the fact that the factors behind the choice of three leading values include all-year-round exercise, the professional character of training manifesting itself in walking with an instructor, and the treatment of pole walking as a sports activity. Another factor, education, draws attention to the awareness aspects that imply the choice of the three values discussed and the self-discipline or working on oneself components associated with Nordic Walking. The choice of the last of these values by individuals from Western countries lends strength to the above conclusion if we assume that conscious prevention is particularly their domain. In the context of socialization processes, theses about the socializing role of sport, and questions concerning the contemporary methods of making young people socially active, it should be stressed that for pole walkers belonging to this age category this sport is associated with personal growth as a value. Finally, the focus on health in individuals with average quality of life and financial situation draws attention to the group for whom the significance of Nordic Walking lies in its accessibility and universality combined with observable health potential.

The above findings may inspire social scientists to formulate further research questions concerning the axiological dimension of sports activity and its links with different categories of independent variables. In the future, it is also worth conducting longitudinal research, making it possible to assess the dynamics or stability of attitudes and axiological references among Nordic Walkers in the context of the development of the phenomenon itself. It would also be interesting to perform analyses including values as independent variables and to focus on the participatory aspect of Nordic Walking, which will be addressed in further parts of the report from the current research project. The research results discussed here can be useful for health care experts and for those responsible for building prevention strategies in various social groups. This is because they draw attention to the category of accessible and at the same time effective activities, with high individual and social potential. At the same time, they show that an important element of promotion strategies and social campaigns aimed at popularizing sport and healthy lifestyle should be axiological categories as significant predictors of individuals’ actions and decisions.
